# Effect of the Extracellular Vesicle RNA Cargo From Uropathogenic *Escherichia coli* on Bladder Cells

**DOI:** 10.3389/fmolb.2020.580913

**Published:** 2020-09-25

**Authors:** Priscila Dauros-Singorenko, Jiwon Hong, Simon Swift, Anthony Phillips, Cherie Blenkiron

**Affiliations:** ^1^Department of Molecular Medicine and Pathology, Faculty of Medical and Health Sciences, The University of Auckland, Auckland, New Zealand; ^2^Department of Surgery, Faculty of Medical and Health Sciences, The University of Auckland, Auckland, New Zealand; ^3^School of Biological Sciences, Faculty of Science, The University of Auckland, Auckland, New Zealand; ^4^Surgical and Translational Research Centre, The University of Auckland, Auckland, New Zealand; ^5^Auckland Cancer Society Research Centre, Faculty of Medical and Health Sciences, The University of Auckland, Auckland, New Zealand

**Keywords:** *Escherichia coli*, extracellular vesicles, RNA, bladder cells, lipopolysaccharide

## Abstract

Iron restriction in mammals, part of innate antimicrobial defense, may be sensed as a signal by an infecting pathogen. Iron-dependent regulators not only activate the pathogen’s specific iron acquisition and storage mechanisms needed for survival but also influence a number of other processes. Bacterial extracellular vesicles (EVs) are a conserved communication mechanism, which can have roles in host colonization, transfer of antimicrobial resistance, modulation of the host’s immune response, and biofilm formation. Here we analyze the iron-responsive effect of RNA cargo from *Escherichia coli* EVs in bladder cells. No differences were found in total RNA quantified from EVs released from representative pathogenic and probiotic strains grown in different iron conditions; nevertheless, lipopolysaccharide (LPS) associated with purified RNA was 10 times greater from EVs derived from the pathogenic strain. The pathogen and probiotic EV-RNA have no substantial toxic effect on the viability of cultured bladder cells, regardless of the iron concentration during bacterial culture. Transcriptomic analysis of bladder cells treated with pathogen EV-RNA delivered in artificial liposomes revealed a gene expression profile with a strong similarity to that of cells treated with liposomes containing LPS alone, with the majority being immune response pathways. EV-RNA from the probiotic strain gave no significant perturbation of gene expression in bladder cells. Cytokine profiling showed that EV-LPS has a role modulating the immune response when internalized by bladder cells, highlighting a key factor that must be considered when evaluating functional studies of bacterial RNA.

## Introduction

It is well established that extracellular vesicles (EVs), also known as membrane vesicles, are a conserved communication vehicle across prokaryotes (Chernov et al., 2014; Brown et al., 2015; Schwechheimer and Kuehn, 2015; Erdmann et al., 2017). Regardless of the phylogenetic group of origin, EVs are spherical nanostructures derived from the membrane(s) of parental cells that are released into their extracellular milieu (Gill et al., 2019). Bacterial EVs have been described to carry a diverse molecular cargo (proteins, DNA, RNA, glycolipids, organic small molecules) derived from assorted cellular origins (Nagakubo et al., 2020). Evidence has accumulated to show that bacterial EVs are important signaling packages within the microbial community, which quickly react in response to changes in the environment (Caruana and Walper, 2020). A recent overview of all bacterial EV types has suggested that the two main routes of EV biogenesis, namely, membrane blebbing and cell lysis, strongly influence the EV composition (Toyofuku et al., 2019). The mode of EV biogenesis can be favored by environmental conditions. For example, antibiotics known to cause DNA damage such as fluoroquinolones trigger a SOS response and lyse cells to induce the production of EVs with a double bilayer EV morphology and predominantly cytoplasm-derived protein content (Devos et al., 2017).

When the bacterium’s environment is a mammalian host, EVs are tailored to secure the cell’s survival. In this context, bacterial EVs have been reported with diverse roles in the infection process such as host colonization (Chen et al., 2017), protection against antimicrobials (Chatterjee et al., 2017; Karthikeyan et al., 2020), modulation of the host’s immune response (Davis et al., 2006; Vdovikova et al., 2017), and biofilm formation (Yonezawa et al., 2009; Wang et al., 2015; Grande et al., 2017). Thus, there is a growing need to understand how the host’s cues may specifically impact bacterial EV production and composition, ultimately affecting their biological roles during infection.

Iron is an essential nutrient for many cellular processes of all microorganisms. In the human body and due to its toxicity, most available iron is sequestered, bound to proteins, and therefore restricted as a resource for bacteria (Andrews et al., 2003; Cassat and Skaar, 2013). This scenario in the host, which is part of the innate antimicrobial defense, may be sensed as a signal for the infecting pathogen. Iron-dependent regulators sense iron restriction and activate expression of genes encoding the pathogen’s specific iron acquisition and storage mechanisms needed for survival and at the same time influence a number of other processes, including expression of virulence factors, biofilm formation, acid-shock responses, expression of antigens, and regulation of small RNAs (Dauros-Singorenko and Swift, 2014).

Extracellular Vesicles may also have roles in the process of iron acquisition. For example, in *Mycobacterium tuberculosis*, an iron-restricted environment increases overall EV production along with an EV-mediated iron-scavenging capacity enabling survival of iron-starved cells (Prados-Rosales et al., 2014). In *Pseudomonas aeruginosa*, EVs carrying iron bound to quinolone quorum sensing signals can be scavenged by cells by a Type 6 Secretion System substrate, contributing to survival in iron-depleted environments (Lin et al., 2017). In uropathogenic *Escherichia coli* (UPEC), iron availability in growth conditions can change the biophysical properties of EVs, suggesting modifications in EV composition (Dauros-Singorenko et al., 2017). Moreover, while the quantity of EVs produced by extraintestinal pathogenic *E. coli* is not affected by iron starvation, changes in the EV proteome reflect a stressed cellular state (Chan et al., 2017). Finally, we have seen that iron-replete conditions cause UPEC EVs to be enriched in outer membrane components, while low iron conditions caused no enrichment, suggesting evidence of two different EV biogenesis mechanisms or differential packaging dependent on iron availability (Hong et al., 2019).

Importantly, we identified that UPEC-derived EVs carried proteins enriched in ribosome-related processes, themselves carriers of RNA (Hong et al., 2019). RNA is considered an important and complex communication signal within eukaryotes, prokaryotes, or trans-kingdom (Zeng et al., 2019). Particularly, bacterial small RNAs (sRNA) are reported to modulate gene expression through several mechanisms to control multiple aspects of their life cycle (Storz et al., 2011). Of significant importance to infectious diseases is the utility of RNA-dependent control of virulence factor expression during infection (Quereda and Cossart, 2017). RNA association with EVs has been confirmed in many microorganisms (Sjöström et al., 2015; Blenkiron et al., 2016; Choi et al., 2016). Only a handful of investigations have investigated the functionality of these EV RNAs during the infection on their target host cell with particular roles in subversion of the immune system (Dauros-Singorenko et al., 2018). Examples of such studies include a tRNA fragment identified in *Pseudomonas aeruginosa* EVs demonstrated to reduce EV-induced IL-8 secretion in human bronchial epithelial (HBE) cells (Koeppen et al., 2016) and microRNA-sized RNA fragments identified in periodontal pathogen EVs that reduce IL-5, IL-13, and IL-15 secretion in lymphocytes Jurkat T-cells (Choi et al., 2017). Additionally, periodontal pathogen *Aggregatibacter actinomycetemcomitans* whole EV-RNA cargo induces TNF-α in *in vitro* induced U937 macrophages while also traveling through the blood–brain barrier in mice to induce TNF-α in the brain (Han et al., 2019). Finally, EV-RNA from the foodborne pathogen *Listeria monocytogenes* induces IFN-β in bone marrow-derived macrophages (BMDM), while specific EV-enriched synthesized sRNA species also increase IFN-β in BMDMs and kidney HEK293 cells (Frantz et al., 2019).

Extracellular Vesicles RNA packaging is not however universal. Variations of the RNA composition of EVs in response to different *in vitro* culture conditions have been reported in *Salmonella enterica* Serovar Typhimurium (Malabirade et al., 2018). This study showed that *in vivo* relevant growth conditions to *S. enterica*’s infection life cycle, such as low pH and low phosphate found in the macrophage phagosome or high cell densities that are achieved in the gut extracellular space, induced differential expression of *Salmonella* Pathogenicity Islands in the cell while changing the EV-RNA composition (Malabirade et al., 2018). This highlights the adjustability of the EV-RNA cargo to the growth environment.

Altogether, EV-RNA has potential to be a powerful virulence factor, coupling RNA’s intrinsic variability with the protection, targeting, and delivery granted by EVs. Thus, in this study, we investigated if mimicking the human host’s protective iron-restriction response could influence *E. coli* EV-RNA and any subsequent effects on bladder cells. We show that RNA preparations from bacterial EVs from pathogenic *E. coli* significantly influence transcription in cultured bladder cells, regardless of their iron availability during growth conditions, while non-pathogenic EV-RNA does not. However, the pathogen EV-RNA effect is difficult to differentiate from the effects of LPS co-purifying with RNA preparations, as gene expression and cytokine secretion profiles closely overlap. EV-LPS has a role modulating the immune response when internalized by bladder cells, highlighting a key factor that must be considered when evaluating functional studies of bacterial RNA.

## Materials and Methods

### Strains and Culture Conditions

Uropathogenic *E. coli* (UPEC) strain 536 (O6:K15:H31) (Knapp et al., 1986) and probiotic Nissle 1917 (O6:K5:H1) (Nissle, 1918) were grown to exponential phase in iron-restricted RPMI 1640 medium (R) (Thermo Fisher Scientific) or RPMI supplemented with 10 μM FeCl_3_ (RF), at 37°C with shaking at 200 rpm. The cultures were diluted 1:100 in 2 L of RF or R medium to an optical density (OD) at 600 nm of ∼0.015 and grown to stationary phase for ∼16 h overnight.

### EV Purification and Analysis

Extracellular Vesicles methodology is reported as per the Minimal Information for Studies of Extracellular Vesicles (MISEV) 2018 guidelines (Théry et al., 2018). Bacterial cells were depleted from 2 L of culture broth by centrifuging twice at 7,000 × *g* for 10 min at 4°C. Residual cells and large debris were further removed from the supernatant by filtration using a 0.22-μm PES filter (Merck Millipore). Cell-free supernatants were concentrated using a 100 kDa Vivaflow 200 cassette (Sartorius AG) before pelleting EVs by ultracentrifugation at 75,000 × *g* for 2.5 h at 4°C (Avanti J-30I centrifuge with JA-30.50 Ti rotor, Beckman). EV pellets were resuspended in PBS (Sigma-Aldrich) and filtered using a 0.22-μm PES syringe filter and further concentrated using 100 kDa Vivaspin 500 columns (Sartorius AG). The resulting “crude” EV preparation (400–600 μg of EV protein in 500 μL) was loaded into a Size Exclusion Chromatography (SEC) column (qEVoriginal, 70 nm, IZON) following the manufacturer’s instructions, with thirteen 0.5-mL eluant fractions collected manually. Reproducibility of this method has been previously described (Dauros-Singorenko et al., 2017).

Extracellular Vesicles fractions were quantified for protein content using bicinchoninic acid (BCA) assay (Thermo Fisher Scientific) according to the manufacturer’s instructions. EVs were counted and sized by nanoparticle tracking analysis (NTA) using a NanoSight NS300 system (Malvern Instruments Ltd.). Each sample was diluted 100–10,000 times in PBS, administered at constant flow rate (50 AU) with an automated syringe pump, and recorded in sets of three videos of 30 s each. The data were analyzed using NTA software version 3.0. Camera level varied between 7 and 8, and detection threshold was 5. Each sample was measured in triplicate, and data were combined for analysis.

Each SEC fraction was quantified for protein content and particle counts to determine EV-rich fractions (Fractions 8–10 in Nissle and Fractions 8–11 in UPEC), which were pooled and concentrated with 100-kDa Vivaspin 500 columns. The resulting “pure” EV preparation was stored at −80°C for further use in RNA extraction.

### Lipopolysaccharide (LPS) Quantification

Lipopolysaccharide was quantified by EndoZyme recombinant Factor C Endotoxin Detection Assay (Hyglos GmbH, Germany). The procedure was performed as per the manufacturer’s instructions. Plates were read on a PerkinElmer EnSpire 2300 plate reader, and the results were expressed in endotoxin units (EU) and transformed to nanograms of LPS by the equation 10 EU/mL = 1.0 ng/mL.

### EV-RNA Extraction

Pure bacterial EV preparations were resuspended in TRIzol LS (Thermo Fisher Scientific), 200 μL of chloroform, and 2 μL of 5 mg/mL glycogen (Thermo Fisher Scientific) added. Samples were vortexed for 15 s, incubated for 10 min on ice, and centrifuged at 13,000 × *g* for 10 min at 4°C. The upper aqueous phase was collected and mixed with 1.25 volumes of 100% ethanol, and RNA purified using mirVana RNA isolation kit (Thermo Fisher Scientific), according to the manufacturer’s protocol for total RNA isolation. Cultured 5637 human bladder carcinoma cells in TRIzol (Thermo Fisher Scientific) were mixed with 50 μL of miRNA Homogenate Additive (mirVana RNA isolation kit), 100 μL of chloroform, and 2 μL of 5 mg/mL glycogen. Samples were vortexed and incubated for 10 min on ice to continue with protocol as described above for extractions from bacterial EVs. RNA yields were determined by a Qubit 2.0 fluorometer using Qubit RNA HS assay kit (Thermo Fisher Scientific).

### Viability Assays

Human bladder carcinoma cells (5637 ATCC HTB-9) were maintained with RPMI 1640 medium supplemented with 10% vol/vol fetal bovine serum (Moregate Biotech). For the assay, bladder cells were seeded in tissue culture-treated 96-well plates and incubated at 37°C and 5% CO_2_ to reach a density of 60,000 cells/cm^2^. A concentration range of RNA extracted from bacterial EVs (EV-RNA), UltraPure LPS (*E. coli* O111:B4, InvivoGen), Poly I:C at 0.1 μg/mL (polyinosinic–polycytidylic acid sodium salt, Sigma), or R848 at 1 μg/mL (Resiquimod, InvivoGen) was mixed with transfection vehicle Lipofectamine 2000 (LF, Thermo Fisher Scientific), following the manufacturer’s instructions. EV-RNA/LF mixtures were added onto bladder cells in triplicate wells along with reduced serum medium Opti-MEM (Thermo Fisher Scientific) with a final volume of 200 μL. Control wells were treated with equivalent amounts of LF. At the 4-h viability assessment, the supernatant (SN) was removed and stored for cytokine assays, and 90 μL of fresh growth medium and 10 μL of PrestoBlue (Thermo Fisher Scientific) were added to each well and incubated for 1 h. Fluorescence (excitation/emission 560 nm/590 nm) was read in a plate reader (EnSpire, PerkinElmer). For the 24-h time point, 4 h SN was discarded from wells and replaced with 200 μL of fresh growth media with growth allowed to continue up to 24 h of incubation, whereupon the viability assessment was repeated.

### Transcriptome Analysis

Bladder cells were seeded at a density of 50,000 cells/cm^2^ in tissue culture-treated 24-well plates and incubated at 37°C and 5% CO_2_. Forty-eight hours later, 2 ng/mL of EV-RNA or 1000 ng/mL of LPS mixed with Lipofectamine was added onto triplicate bladder cell wells along with reduced serum medium Opti-MEM with a final volume of 1 mL. Control/untreated wells contained cells treated with PBS. After incubation for 4 h, SN was removed, and 500 μL of TRIzol was added to adherent cells, resuspended, and stored at −80°C until RNA extraction. Transcriptomic profiling of RNA from treated bladder cells was performed using Affymetrix Clariom S Human GeneChip arrays (Thermo Fisher Scientific) as a contract service by the Auckland Genomics facility. Quality control, normalization, and differential gene expression analysis was performed using Transcriptome Analysis Console (TAC) Software Version 4.0.2.15 (Thermo Fisher Scientific). Analysis of variance across all conditions was performed with ANOVA *F*-test, and false discovery rate (FDR) *p*-value < 0.0001 was considered significant. Differential gene expression in pairwise comparisons (compared to untreated) were included as significant with fold changes (FC) > 2 or −2, *p* < 0.05 and *t*-test with FDR *p* < 0.05. Array data has been deposited in the GEO database^1^ accession number GSE148711.

### Pathway Analysis

Pathway analysis was performed with upregulated genes from pairwise comparisons in PANTHER http://www.pantherdb.org/(Mi and Thomas, 2009; Mi et al., 2019) version 14.1 from the 2018_04 release of the Reference Proteome dataset. The following selections were used in the analysis: PANTHER Overrepresentation Test (Released 20190711); Annotation Version and Release Date: Reactome version 65 Released 2019-12-22; Reference List: Homo sapiens (all genes in database); Test Type: Fisher’s Exact with FDR *p* < 0.05.

### Cytokine Profiling

Cytokine quantification on cell culture SN from viability assays was performed using a human cytokine/chemokine magnetic bead panel, premixed with 29-plex immunology assay in a 96-well plate (MILLIPLEX MAP). The assay was performed according to the manufacturer’s instructions, and the plate was read and analyzed with a Luminex MAGPIX instrument.

## Results

### Iron Culture Conditions Do Not Determine EV RNA Total Content

Extracellular Vesicles from pathogenic UPEC 536 (UPEC) and probiotic Nissle 1917 (Nissle) *E. coli* strains were purified from 3 independent replicate cultures. Each EV crude preparation was purified by SEC and resulting fractions quantified in protein and particle counts to demonstrate EV enrichment in fractions (Dauros-Singorenko et al., 2017). EV morphological analysis on these types of samples by transmission electron microscopy has been previously described (Hong et al., 2019).

**FIGURE 1:**
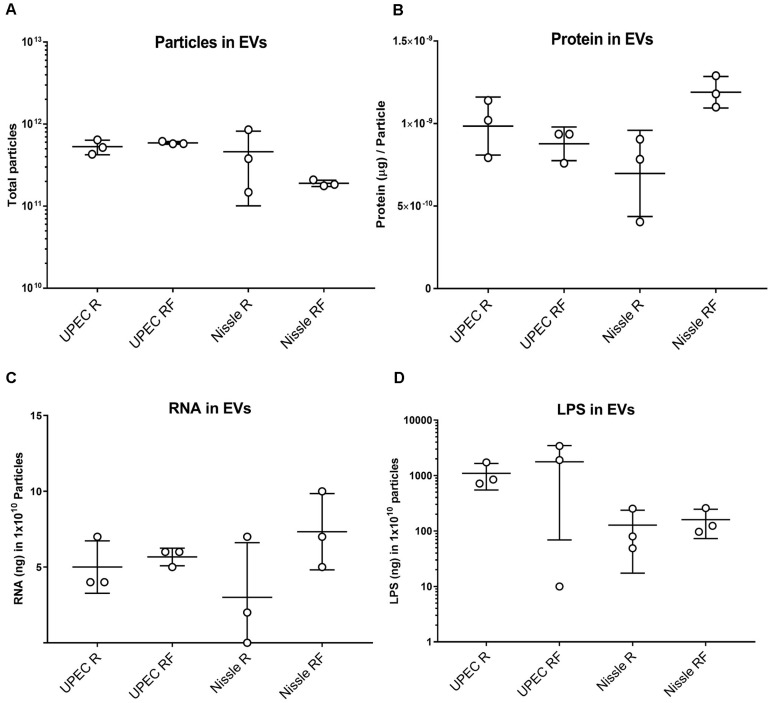
Comparison of molecular content in SEC-purified EVs isolated from UPEC and Nissle grown with different iron availabilities (low iron R and high iron RF). **(A)** Total particles in EV preparations. **(B)** Protein content in EV preparations (μg of protein per particle). **(C)** RNA content in EV preparations (ng of RNA in 10^10^ particles). **(D)** LPS content in EV preparations (ng of LPS in 10^10^ particles). Data showing mean and standard deviation from three biological replicates.

There were no significant differences in the overall particle count or protein content of EVs isolated from either UPEC or Nissle, grown in iron starved (R) or iron replete (RF) conditions (Figures 1A,B). Similarly, the RNA concentration of EVs showed no significant differences between strains or iron conditions (Figure 1C).

Extracellular Vesicles from Gram-negative bacteria constitutively harbor LPS (Ellis and Kuehn, 2010); thus, we questioned if RNA from EVs could co-isolate with LPS. LPS was indeed found in all EV-RNA preparations. No differences were found in LPS from EV-RNA preparations between cells grown in two iron conditions (Figure 1D), although UPEC EV-RNA contained 10 times more LPS than Nissle EV-RNA (UPEC EV-RNA LPS mean of 263 ng of LPS/ng of RNA, Nissle EV-RNA LPS mean of 24 ng of LPS/ng of RNA).

### Bacterial EV-RNA Is Not Cytotoxic to Bladder Cells

**FIGURE 2:**
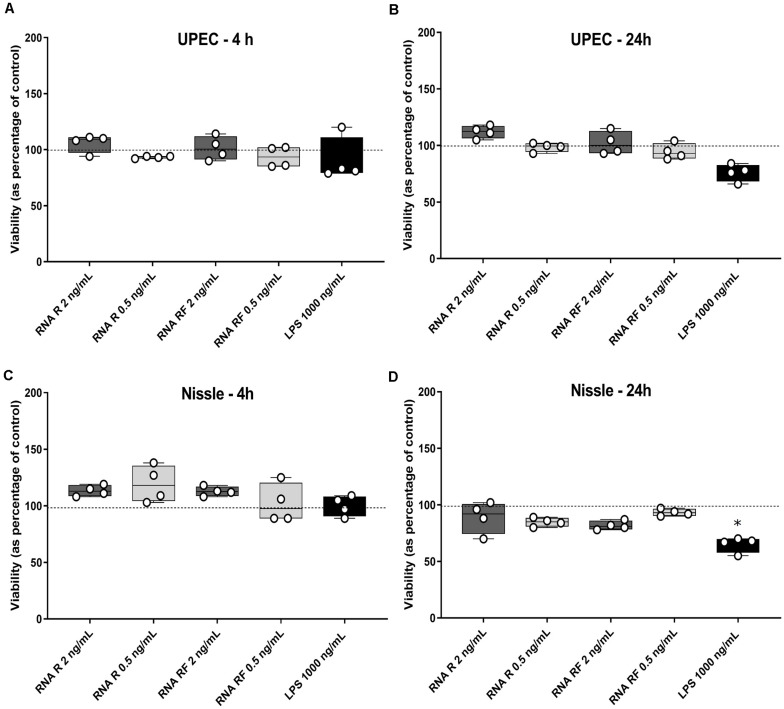
EV-RNA is not cytotoxic to cultured bladder cells. Viability of cultured bladder cells treated with UPEC and Nissle EV-RNA for 4 h **(A,C)** and 24 h **(B,D)**. Viability shown as percentage of control (transfection agent only in dotted line). Data plotted as box and whiskers (box represented by upper and lower quartile, line = median, bars = min and max values) of 4 replicates (circles) and stars denote significant differences compared to control (transfection agent only) determined by one-way ANOVA Kruskal–Wallis and Dunn’s posttest (*p* < 0.05).

We next set out to determine the phenotypic effect of bacterial EV-RNA on mammalian cells, by analyzing the viability of cultured bladder cells after treatment. To mimic EV-mediated entry into cells, RNA was mixed with a liposome-forming transfection agent prior to treatment. Previously, we have shown that UPEC EVs are quickly taken up by bladder cells (within 2 h) and 1% of delivered EV-RNA can be detected in the same time frame (Blenkiron et al., 2016). In line with this, two concentrations of EV-RNA were tested (dose of 0.5 ng or 2 ng EV-RNA/mL) in order to determine and confirm a non-toxic dose and time point for further studies. At 4 and 24 h posttreatment, there was no significant difference in the viability of cells treated with either dose of EV-RNA from UPEC or Nissle irrespective of whether the cultures from which the EVs were purified were grown in iron-sufficient (RF) or iron-restricted (R) conditions (Figures 2A–D). LPS (1000 ng/mL) showed no cytotoxic effect at 4 h but gave a pronounced effect on cell viability in Nissle at 24 h, averaging 30–40% reduction in viability when compared to control. These results suggest that overall, there is no effect on cell viability from EV-RNA. Consequently, we chose 2 ng/mL as a non-cytotoxic treatment dose and 4 hours as the time point for further early response experiments.

### Transcriptomic Response of Bladder Cells to RNA Isolated From Bacterial EVs

To investigate the early effects of EV-RNA on bladder cells, transcriptome analysis was performed on bladder cells treated for 4 h with 2 ng/mL of EV-RNA from UPEC 536 (U) and Nissle 1917 (N) grown in iron-sufficient (RF) and iron-restricted (R) media. For comparison, bladder cells were challenged with LPS at two doses, 1000 ng/mL (HL) and 8 ng/mL (LL). HL and LL treatment doses were chosen based on the highest and lowest LPS contents found in RNA preparations of UPEC EV-RNA and Nissle EV-RNA, respectively.

**FIGURE 3:**
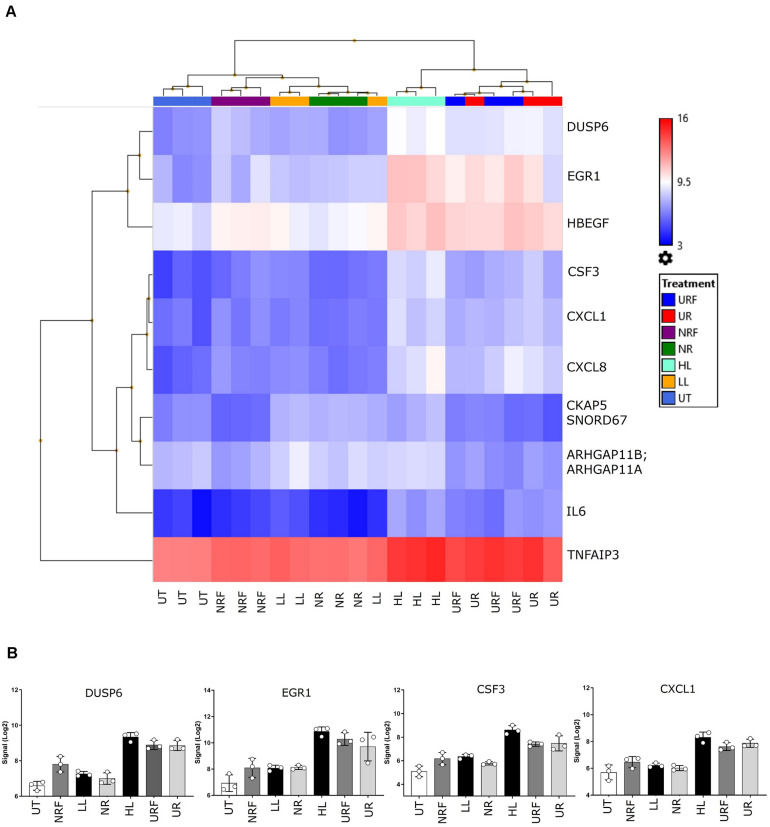
Transcriptional response of bladder cells to bacterial EV-RNA. **(A)** Unsupervised clustered heat map of analysis of variance in expression array data from bacterial EV-RNA or LPS-treated bladder cells selected for the most significantly regulated genes across all conditions (ANOVA *p* < 0.05, FDR *p* < 0.0001) on the *Y*-axis and all treatments on the *X*-axis (*n* = 3). Color scale indicates intensity of signal (Log2) in arbitrary range, blue gradient < 9.5 < red gradient. **(B)** Gene expression of selected genes across all treatments, intensity of signal (Log2) on the *Y*-axis and all treatments on the *X*-axis. Data plotted as mean and SD of 3 replicates. HL, high dose of LPS (1000 ng/mL); LL, low dose of LPS (8 ng/mL); NR, Nissle EV-RNA from iron-starved conditions; NRF, Nissle EV-RNA from iron-replete conditions; UR, UPEC EV-RNA from iron-starved conditions; URF, UPEC EV-RNA from iron-replete conditions; UT, untreated PBS control.

Firstly, unsupervised clustering was performed on all samples using the most significantly differential genes across all conditions (ANOVA *F*-test FDR *p* < 0.0001). Clustering using expression levels of these highly variable genes identified two main groups: Cluster 1, UPEC RF EV-RNA (URF), UPEC R EV-RNA (UR), high-dose LPS (HL) and Cluster 2, Nissle RF EV-RNA (NRF), Nissle R EV-RNA (NR), low-dose LPS (LL), and untreated (UT) (Figure 3A). Selected genes with the most significant FDRs are shown in Figure 3B, where a high gene expression in treatments with UR and URF EV-RNA is evident and also seen in the High LPS treatment. A low dose of LPS (LL), a treatment selected due to the average LPS amount found in Nissle EV-RNA, consistently showed gene expression similar to Nissle EV-RNA-treated cells. From literature review, it is clear that the most significantly differential genes across conditions are involved in immune response (Table 1). Upregulation of common genes such as *EGR1* and *CXCL8* in HL, UR, and URF conditions suggests that this effect may be in part due to the higher levels of LPS contamination in UPEC EV-RNA samples than in Nissle EV-RNA.

**TABLE 1:** Differentially expressed genes driving grouping of the two distinctive treatment clusters.

Gene	FDR p-value	Function
*DUSP6*	1.09E-06	Dual-specificity phosphatase 6; inactivates ERK1/2 in cytoplasm, which mediates cell division, migration, and survival
*CSF3*	3.53E-06	Granulocyte-colony-stimulating factor (G-CSF); stimulates the survival, proliferation, differentiation, and function of neutrophils.
*CXCL8*	6.63E-06	Chemokine (C–X–C motif) ligand 8 (interleukin 8), proinflammatory cytokine recruits and activates neutrophils during inflammation
*CXCL1*	6.63E-06	Chemokine (C–X–C motif) ligand 1 (GROA); induces neutrophil release from the bone marrow and recruits them to inflammation site
*IL6*	9.41E-06	Interleukin 6, pro-inflammatory cytokine inducing synthesis and secretion of acute-phase proteins by the liver
*EGR1*	1.90E-05	Early growth response protein 1, transcriptional regulator (activator) of genes involved in differentiation and mitogenesis
*HBEGF*	4.38E-05	Heparin-binding EGF-like growth factor, involved in cell growth and tumor progression
*TNFAIP3*	4.38E-05	Tumor necrosis factor alpha-induced protein 3; inhibits NF-kappa B activation, limiting inflammation
*CKAP5; SNORD67*	5.63E-05	Cytoskeleton-associated protein 5 (CKAP5), roles in spindle formation during mitosis; small nucleolar RNA (SNORD67), modifies other snRNAs which help in processing pre-mRNA
*ARHGAP11A; ARHGAP11B*	6.81E-05	ARHGAP11A encodes a Rho GTPase-activating-protein; ARHGAP11B (truncated version of ARHGAP11A), does not show RhoGAP activity

### Iron-Mediated Effect of Bacterial EV-RNA on Host Cell Transcriptional Pathways

**FIGURE 4:**
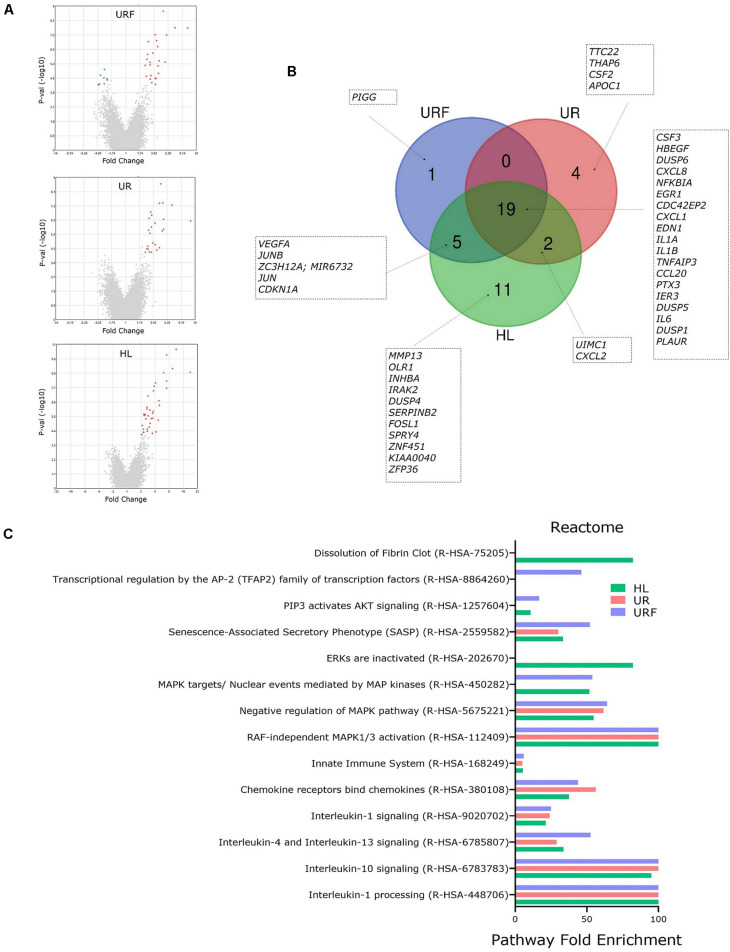
Pathway analysis of regulated genes in bladder cells treated with iron-responsive UPEC EV-RNA. **(A)** Volcano plots of all regulated genes in bladder cells in response to UPEC EV-RNA or LPS treatments (compared to untreated control). **(B)** Venn diagram with details of upregulated genes in bladder cells in response to UPEC EV-RNA or LPS treatments (compared to untreated control). **(C)** Pathway analysis performed by PANTHER Overrepresentation Test, using Reactome annotation dataset in upregulated genes in bladder cells in response to UPEC EV-RNA or LPS treatments (compared to untreated control). Enriched pathways (FDR *p* < 0.05) are shown in *Y*-axis and fold enrichment in *X*-axis. UR, UPEC EV-RNA from iron-starved conditions; URF, UPEC EV-RNA from iron-replete conditions; HL, high dose of LPS (1000 ng/mL).

For a detailed exploration of effects of EV-RNA on bladder cell gene expression, we performed pathway analysis on all regulated genes from selected pairwise comparisons (FC > 2 or −2 and *t*-test FDR *p* < 0.05) using PANTHER (Mi and Thomas, 2009; Mi et al., 2019). Comparisons of UPEC EV-RNA treatments UR, URF, or HL individually to the untreated (UT) revealed 25, 25, and 37 upregulated genes, respectively (Figure 4A). Only URF had significantly downregulated genes compared to untreated (8 genes). From the diagram in Figure 4B, we can see that most upregulated genes (19) are shared between the three treatments, with one gene being unique to URF and 4 to UR. These results corroborate that a large part of the effect of pathogenic UPEC EV-RNA may be attributed to its LPS content, more than any effect from the bacterial growth conditions and differential EV-RNA packaging.

Pairwise comparisons of Nissle EV-RNA, R, and RF to the UT showed no significantly regulated genes with a FDR *p* < 0.05; therefore, no pathway analysis could be performed. This suggests that the overall RNA cargo from Nissle EVs does not elicit a significant transcriptomic response in bladder cells compared to the normal growth course of these cells.

Reactome pathway analysis in cells treated with UPEC EV-RNA or LPS revealed that there is a significant overlap of pathways induced by these treatments (Figure 4C). Pathways such as inactivation of “Interleukin-1 processing,” “Interleukin-10 signaling,” “Interleukin-4 and Interleukin-13 signaling,” “Interleukin-1 signaling,” “Chemokine receptors bind chemokines,” and “Innate Immune System” are strongly enriched due to shared upregulation of cytokine genes *IL6*, *CXCL8*, *IL1A*, *IL1B*, *CCL20*, *CXCL2*, and *CXCL1*. A second subset of enriched pathways is shared among the three treatments UR, URF, and HL: “RAF-independent MAPK1/3 activation” and “Negative regulation of MAPK pathway.” Upregulated genes of dual-specificity phosphatases (*DUSP1*, *DUSP4*, *DUSP5*, and *DUSP6*) are responsible for inactivating the mitogen-activated protein kinase (MAPK) pathway, which communicates extracellular signals to gene expression changes affecting cell-cycle progression and survival (Lang and Raffi, 2019). Another extracellular signal pathway “PIP3 activates AKT signaling” is overrepresented only in HL and URF treatments, mainly due to upregulation of genes *JUN*, *EGR1*, *CDKN1A*, *IER3*, and *HBEGF*, whereas the enrichment of pathway “Dissolution of Fibrin Clot” is unique to HL treatment.

### Effect of EV-RNA on Host Secretome

**FIGURE 5:**
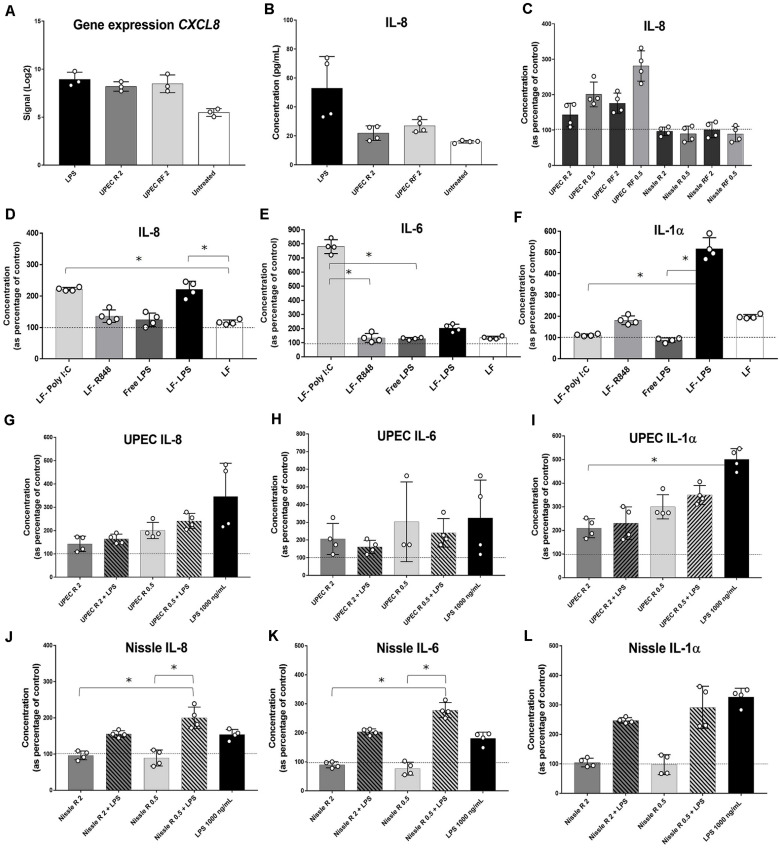
Cytokine profiling in bladder cells in response to EV-RNA. **(A)** Gene expression of *CXCL8* assessed by microarray. **(B–F,H–L)** bladder cells treated with different treatments for 4 h, after which supernatants were assessed by Luminex assays. UPEC, EV-RNA from UPEC strain; Nissle, EV-RNA from Nissle strain; R, EV-RNA obtained from iron-limiting culture conditions; RF, EV-RNA obtained from iron-sufficient culture conditions; 2, EV-RNA at 2 ng/mL; 0.5, EV-RNA at 0.5 ng/mL; LPS, LPS at 1000 ng/mL delivered with transfection agent unless stated, Free-LPS; LF-Poly I:C, Poly I:C delivered with transfection agent; LF-R848, Resiquimod R848 delivered with transfection agent. Data is plotted as mean and SD of 4 replicates, concentration in *Y*-axis as percentage of control (transfection agent only in dotted line in **C**,**G–L**; PBS only in dotted lines in **D–F**). Stars denote significant differences between treatments determined by one-way ANOVA Kruskal–Wallis and Dunn’s posttest (*p* < 0.05).

In order to determine the early EV-RNA effect on cellular immune responses, cytokine secretion assays were performed on conditioned media from EV-RNA-treated bladder cells after 4 h of treatment. Firstly, we investigated whether the effects of EV-RNA on IL-8 are caused solely by the contaminant LPS cargo. Comparison of gene expression and cytokine secretion data from bladder cells treated with UPEC EV-RNA or LPS revealed that at 4 h *CXCL8* transcripts increase, to a similar extent, in response to LPS or EV-RNA, regardless of the iron culture conditions (Figure 5A). Conversely, while IL-8 cytokine secretion was induced at 4 h after LPS challenge, there was no substantial response to UPEC EV-RNA (Figure 5B).

Next, investigating the dose response of host cells to EV-RNA from different strains, we observed that secretion of IL-8 was inversely proportional to the amount of EV-RNA from UPEC, i.e., 0.5 ng/mL of EV-RNA induced secretion of 1.6 × more IL-8 protein than when cells were treated with 2 ng/mL EV-RNA (Figure 5C). This effect was seen for EV-RNA isolated from UPEC in both iron growth conditions. Treatment with Nissle EV-RNA did not however induce IL-8 secretion (Figure 5C).

Next, we characterized secretion of pro-inflammatory cytokines, by treating cells with a known TLR3 dsRNA-response inducer Poly I:C (Alexopoulou et al., 2001), a TLR7/8 ssRNA-response inducer R848 (Jurk et al., 2002), 1000 ng/mL of LPS delivered to culture media (free LPS), or 1000 ng/mL of LPS delivered to the cytoplasm with Lipofectamine (LF-LPS). Figure 5D shows that both Poly I:C and LF-LPS induce IL-8 (and GM-CSF, Supplementary Figure S1A) secretion to a similar range, unlike R848 which is unaltered from control. Cytokines IL-6 (and TNF-α, Supplementary Figure S1B) and IL-1α are strongly induced preferentially by Poly I:C and LPS, respectively (Figures 5E,F), when cell viability at the time of cytokine assessment was similar for all treatments (Supplementary Figures S1C,D). INF-α2 levels were below the sensitivity of the assay for all treatments (data not shown). It is worth noting that extracellular free LPS and R848 did not trigger any cytokine secretion in bladder cells, suggesting that TLR4 LPS cell surface receptor and TLR7/8 ssRNA receptor are somehow not responsive in these bladder cells.

Since cellular transcriptomic and cytokine analyses revealed no substantial differences between the effect of EV-RNA from R and RF conditions (Supplementary Figure S1E), from here on we present the effects of EV-RNA cargo secreted under iron-limiting R conditions, as the most relevant growth scenario in the host (Robinson et al., 2018).

One hypothesis for the role of EV-RNA in pathogenesis would be to dampen the immune response that is triggered by other secreted bacterial components (Koeppen et al., 2016; Choi et al., 2017). To investigate this, we treated cells with EV-RNA (0.5 or 2 ng/mL), LPS (1000 ng/mL), or a combination of EV-RNA and LPS delivered to the cytoplasm using Lipofectamine. We hypothesized that the EV-RNA would reduce cytokine secretion initiated by LPS when treated in concert. A dose of 2 ng/mL of UPEC EV-RNA supplemented with LPS was found to reduce IL-8 and IL-1α secretion, in average by 53 and 54%, respectively, when compared to the same dose of LPS alone, suggesting that internalization of UPEC EV-RNA cargo in bladder cells suppresses a simultaneously induced LPS response (Figures 5G,I). In contrast, the dsRNA-induced response is unaltered by cotreatment with LPS and UPEC EV-RNA, characterized by a stable IL-6 and TNF-α secretion throughout all treatments (Figure 5H, Supplementary Figure S1F).

For Nissle EV-RNA, induction of IL-8, IL-6, and IL-1α secretion was only seen when LPS was added (Figures 5J–L, respectively). TNF-α, in turn, remained unchanged regardless of LPS addition (Supplementary Figure S1G), becoming apparent that IL-6 and TNF-α are differentially regulated by Nissle EV-RNA. Addition of LPS to 2 ng/mL Nissle EV-RNA caused a smaller reduction (∼24%) of IL-1α secretion compared to LPS alone, suggesting that the dampening of IL-1α LPS-induced response in presence of EV-RNA might be modulated by the concentration of native LPS found in EV-RNA.

## Discussion

Extracellular Vesicles structure in Gram-negative bacteria is largely determined by their biogenesis mechanism. EVs generated by blebbing of the outer membrane (OM) are enriched in outer membrane components, including large quantities of LPS. In these OM-derived EV types, LPS can be highly abundant in a 10:1 ratio compared to the protein content (Ellis and Kuehn, 2010). EVs generated by an explosive lysis mechanism will contain outer and inner membrane components, and cytosolic ones, such as RNA (Toyofuku et al., 2019). As significant amounts of LPS were found in RNA extractions from EVs, we highly recommend that LPS in EVs should not be overlooked, especially its persistence in samples when investigating other molecules of interest, such as our RNA extractions. Many LPS extraction methods overlay with RNA extraction methods (Johnson and Perry, 1976), even with the use of ready-made RNA-extracting commercial reagents (Yi and Hackett, 2000). Native EVs in an infection carry both RNA and LPS; therefore, their contribution to an effect in the host should be analyzed conjointly.

Differences in the amount of EV-associated LPS were identified here between *E. coli* strains, regardless of pathogenic UPEC 536 and non-pathogenic Nissle 1917 belonging to the same O6 serotype. This effect has been previously reported between pathogenic and non-pathogenic *E. coli* EVs (Behrouzi et al., 2018). Pathogens, such as UPEC, have evolved to succeed in infection, with mechanisms of LPS modification that work toward immune system evasion (Maldonado et al., 2016), ultimately affecting the LPS load in EVs. Although strain differences were seen, we did not find differences in total quantification of LPS content in EVs secreted under iron restriction compared to iron sufficient conditions in both strains. It is interesting to note, however, that differences in the LPS composition, with shorter O-chains, has been reported in *Helicobacter pylori* EVs released under iron restriction (Keenan et al., 2008), as well as other culture conditions being able to remodel the LPS structure and stability (Needham and Trent, 2013; Sharp et al., 2019). It is unclear to us the mechanistic nature of differences of LPS quantity in EVs between strains used in this study; nevertheless, these differences may have a great impact on the EV uptake by the host cell (O’Donoghue et al., 2017).

From analysis of transcriptomic data in the host cell when exposed to EV-RNA from different strains or two doses of LPS, we could see that probiotic Nissle strain EV-RNA had a minimal effect on cultured bladder cells, and this was similar to the low dose of LPS and untreated cells. It has been reported that Nissle 1917 EVs are taken up; induce secretion of IL-6, IL-8, and TNF-α; and prevent enteropathogenic *E. coli* (EPEC)-mediated barrier disruption in Caco-2 intestinal epithelial cells (Fábrega et al., 2016; Alvarez et al., 2019). It is clear in our study that Nissle 1917 EV-associated RNA cargo is not however involved in any significant transcriptional or cytokine secretion by bladder cells. The most significant changes in gene expression were elicited by pathogen UPEC 536 EV-RNA and a high dose of LPS (1000 ng/mL), clustering together in the heat map from the microarray data. In this study, treatment of 5637 bladder cells with pathogen EV-RNA or high-dose LPS lead to a unique early response cytokine profile characterized by increased gene expression of *IL6*, *CXCL8*, *IL1A*, *IL1B*, *CCL20*, *CXCL2*, and *CXCL1.* All these genes have many layers of transcriptional regulation reflected in part in their promoter regions containing binding sites for numerous transcription factors, such as AP-1, NF-IL6, SP1, and CREB. More importantly, all the aforementioned genes are subject to control of NF-κB, one of the most important transcription factors, central to the pro-inflammatory response in cells (Liu et al., 2017).

Pathway analysis of regulated genes in pairwise comparisons with untreated controls revealed, again, that treatments of high dose of LPS or UPEC 536 EV-RNA induce predominantly the same pathways, falling into two categories: regulation of extracellular signal transduction and secretion of effector cytokines. The regulation of an immune response is complex, where the duration and intensity are tightly regulated and may be substantially dissimilar in different cell types. Dual-specificity phosphatases (DUSPs) are involved in aspects of balancing the response of extracellular signal kinase-mediated pathways, mainly ERK, JNK, and p38 pathways (Ahmad et al., 2018). While specific ligands can induce specific DUSPs, also the same stimulus can generate different DUSP expression responses depending on cell type (Lang and Raffi, 2019). Here, we found that 5637 bladder cells increase expression of *DUSP1*, *DUSP4*, *DUSP5*, and *DUSP6* when challenged with UPEC EV-RNA or LPS. It has been previously reported that DUSP1, DUSP4, and DUSP5 are induced by LPS, and DUSP1, DUSP5, and DUSP6 negatively regulate their corresponding kinase-substrates (Lang and Raffi, 2019). Other DUSPs (e.g., DUSP11) have an affinity for triphosphorylated RNA substrates (bacterial and viral RNA) (Lang and Raffi, 2019); however, we did not find these upregulated.

The immune response to LPS has been extensively investigated; however, it is still far from a complete understanding. Toll-like receptor 4 (TLR4) is the most important cell-surface LPS-sensing mechanism, but it involves many accessory molecules that vary in presence or abundance between species (mouse or human) and among cell types (immune or non-immune cells). Lipid A-binding protein (LBP), CD14, MD-2, and TLR4 have distinctive roles in recognizing extracellular LPS, with the signal activating two main pathways: MYD88 or TRIF (Steimle et al., 2016). The MYD88 pathway recruits several kinases (e.g., MAPK, IKK), which then cascades into activation of transcription factors AP-1 and NF-κB, leading to upregulation of pro-inflammatory cytokines such as IL-6, IL-18, IL-1β, and tumor necrosis factor (TNF) (Lu et al., 2008). Endocytosis of TLR4 triggers the TRIF pathway activating transcription factor IRF3 and late-phase activation of NF-κB, resulting in upregulation of type-I interferon (IFN) (Rosadini and Kagan, 2017). Intracellular LPS-sensing occurs through the cytosolic multiprotein complex NLRP3 inflammasome, requiring two independent events (Steimle et al., 2016; Rathinam et al., 2019). Firstly, a TLR-mediated signal must induce the expression of inflammasome members and translation of inactive pro-IL-1β and pro-IL-18 pro-inflammatory cytokines. Secondly, caspase 4 or 5 binds directly to LPS in the cytosol, with the subsequent activation of the NLRP3 inflammasome and pro-IL-1β and pro-IL-18 cleavage by caspase 1, leading to pyroptosis death (Diamond et al., 2015; Barker and Weiss, 2019). In this study, transcription of *IL1B* was increased in bladder cells exposed to UPEC 536 EV-RNA or LPS; however, *NLRP3* transcripts were unaffected by these treatments.

RNA-sensing occurs through intracellular endosome receptors. TLR3 detects dsRNA and signals via the TRIF pathway, whereas TLR7/8 detects dsRNA and activates the MYD88 pathway (Heil et al., 2004; Biondo et al., 2012). There is also accumulating evidence of activation of NLRP3 inflammasome by bacterial RNA in immune cells (Sander et al., 2012; Sha et al., 2014; Vierbuchen et al., 2017); however, there is no defined cytosolic RNA-binding receptor directly linked with the inflammasome. In this study, there were no genes exclusively induced by EV-RNA, suggesting that the LPS content in our EV-RNA samples is fundamental in overall responses seen in bladder cells to UPEC 536 EV-RNA.

The majority of receptor-induction, signaling pathways and effector outcomes have been deciphered in immune cells and show that the choice and intensity of the immune response depends on the specific cell type, making many reports not directly translatable to the scenario seen in, for example, epithelial cells. Here, characterization of the response of cultured bladder cells to known RNA inducers showed us a differential response to RNA mimics or LPS. We saw that dsRNA (Poly I:C, TLR3) strongly induces IL-6 and TNF-α, while ssRNA (R848, TLR7/8) and LPS do not. This is in contrast to reports of other similar cell lines, intestinal Caco2 or kidney 293T, which are not responsive to Poly I:C (Alexopoulou et al., 2001). In macrophages, transfected dsRNA binds to TLR3, with subsequent NF-κB activation and secretion of IL-6, IL-12, TNF, and type 1 IFN (Alexopoulou et al., 2001). We found negligible amounts of IFN-α2, a type I IFN, released from treated bladder cells, suggesting no activation of transcription factor IRF3. These characterizations suggest that 5637 cells are inherently responsive to TLR3-dsRNA-NF-κB-IL-6/TNFa but not to TLR7/8-ssRNA-IRF3-IFN signaling.

When we sought to analyze the dynamic of LPS sensing by the TLR4 surface receptor, we saw that 1000 ng/mL of LPS delivered to extracellular space did not induce any cytokine secretion in 5637 bladder cells at 4 h of treatment. This outcome is in contrast with other studies where 5637 bladder cells express TLR4 accessory molecule CD14, their transcription factor NF-κB is active, and 6-h treatments with 1000 ng/mL of LPS or 3-h treatments with 5000 ng/mL of extracellular LPS increased IL-6 secretion significantly (Schilling et al., 2003; Shimizu et al., 2004; Hunstad et al., 2005). Discrepancies between this and the published studies may be due to different LPS used in the assays, *E. coli* 0111:B4 and *E. coli* O55:B5, respectively.

We observed that cytosolic Lipofectamine delivered LPS strongly induced IL-1α in cultured 5637 bladder cells (more than 400% of control) and mildly induced IL-8 but had no effect on IL-1β, IL-6, and TNF-α secretion. Increased *IL1A* gene expression was seen in cells treated with LPS or UPEC 536 EV-RNA; however, IL-1α secretion into supernatant was only increased by transfected LPS, suggesting posttranscriptional control. TLR4-mediated extracellular LPS stimulation leads to increased secreted IL-1α only when accompanied by NLRP3 inflammasome activation (Fettelschoss et al., 2011); thus, it is unknown to us fully how cytosolic LPS-sensing can induce significant amounts of secreted IL-1α in 5637 cells.

Secreted IL-1β is a strong signature cytokine for NLRP3 inflammasome activation by cytosolic LPS sensing, with a critical role for the development of acute cystitis (Ambite et al., 2016). LPS inflammasome activation has been mainly investigated in immune cells (Vanaja et al., 2016; Santos et al., 2018); however, some non-immune cells may also have this machinery, mostly depending whether caspase 4 is expressed or not (Shi et al., 2014). Specifically, 5637 bladder cells are known to have a responsive NLRP3 inflammasome, as they significantly increase IL-1β secretion when challenged with live *E. coli* UPEC, not commensal strain *E. coli* K-12 (Demirel et al., 2018). Thus, the lack of IL-1β secretion upon treatment with LPS or EV-RNA suggests that neither is able to induce early inflammasome activation in 5637 bladder cells. Guanylate-binding proteins (GBP), important in leaking phagosome content into cytosol, are induced by type 1 IFN (Santos et al., 2018), a cytokine which is absent in SNs from our LPS-treated bladder cells. This information is in accordance with our transcriptomic data, where *IL1B* was induced by LPS alone, possibly due to activation of NF-κB (Di Paolo and Shayakhmetov, 2016), but lacked secretion of the active protein IL-1β, which requires NLRP3 inflammasome-mediated cleavage (Steimle et al., 2016).

Our previous investigations into proteome of EVs showed that UPEC 536 EVs are enriched in rRNA-binding proteins in contrast to Nissle EVs, which are enriched in proteins involved in glycolytic process and ligase activity, leading us to believe that differential packaging of RNA may be causing some of the differences seen between UPEC and Nissle EV-RNA (Hong et al., 2019). A key investigation of this study was to delineate the effects of *E. coli* EV-RNA on host cell responses. UPEC EV-RNA is enriched for rRNA but carries all forms of RNA types (Blenkiron et al., 2016), likely in both ss and dsRNA forms. Bacterial rRNA has been shown to induce IFN in a TLR7-dependent way (Eberle et al., 2009); however, 5637 bladder cells have been found in this study to not be responsive to TLR7/8-ssRNA, and therefore, the use of this cell line may not be fully representative of all cell types.

We hypothesized that culturing bacteria in iron-replete and iron-starved conditions may influence the EV-RNA cargo in a way that would impact transcriptional responses when delivered to cultured bladder cells. Our results did not support this hypothesis, showing no difference in the immune responses of bladder cells when challenged with bacterial total EV-RNA produced under different iron culture conditions. These findings do not invalidate the notion that there might be substantial differences in the RNA composition of EVs isolated from cultures grown in iron-starved and iron-replete conditions. However, in order to investigate this, qualitative assays of greater depth are necessary, such as RNA sequencing.

There are limited publications about the effect of probiotic bacterial RNA on host cells. Transfected mRNA, tRNA, and rRNA from *E. coli* strain ATCC25922 (non-pathogenic) were shown to activate NLRP3 inflammasome and induce IL-1β secretion in human macrophages, an effect independent of double-strand and secondary RNA structure or the presence of 5′-end triphosphate moieties (Sha et al., 2014). Moreover, transfected non-pathogenic *E. coli* total RNA into hepatic stellate cells also induces the NLRP3 inflammasome (Wang et al., 2016).

Combination of treatments of LPS and EV-RNA allowed us to unveil some particular responses in bladder cells. EV-RNA cargo suppresses an LPS-induced IL-1α response, suppression which is proportional to the amount of native LPS in EV-RNA preparations. Since UPEC releases more LPS in their EVs than Nissle, this trait might be an additional virulence advantage.

Although the transcriptome data suggested that much of the early transcriptional response may be confounded by LPS in the EV-RNA, the finding that cotreatment of bladder cells with LPS and UPEC EV-RNA depressed IL-1α secretion is of interest. This suppression response could be explained by development of LPS tolerance in 5637 bladder cells. This is supported by findings of live pathogenic UPEC strains UT189 and NU14 suppressing IL-6 secretion to non-pathogenic *E. coli* K-12 in 5637 bladder cells, an effect which depends on UPEC LPS structure (Hunstad et al., 2005). However, LPS tolerance, shown *in vitro* in immune cells and 5637 bladder cells, leading to subsequent suppression of cytokine TNF-α or IL-6, so far has required prolonged LPS pretreatments and a TLR4-dependent route, as LPS is delivered in extracellular space (Seeley and Ghosh, 2017; Schilling et al., 2003). There is no information about LPS tolerance induced by cytosolic LPS sensing mechanisms nor IL-1α involvement. However, it appears that the ultimate goal of LPS tolerance is to help host cells resist following infections (Seeley and Ghosh, 2017); thus, the EV-LPS-induced response may have important biological consequences in the infection outcome.

Moreover, both dsRNA-induced IL-6 and TNF-α responses were unchanged for UPEC EV-RNA/LPS treatments and dissimilar for Nissle EV-RNA/LPS. These results corroborate that IL-6 and TNF-α have different regulatory networks under low levels of transfected LPS in 5637 bladder cells. Other authors have previously reported a possible defect in the MYD88/IRAK pathway in 5637 bladder cells, since tolerance to extracellular LPS affects IL-6 and not TNF-α secretion, both transcriptionally regulated by NF-κB (Schilling et al., 2003).

Beyond the challenges with appropriate selection of an active recipient cell line, our study might have limitations regarding a relatively low amount of EV-RNA used throughout all the experiments (2 ng/mL). Our purified EV preparations yielded a mean of 350 ng RNA from 2 L of bacterial cultures, meaning a 0.4-ng EV-RNA treatment dose for bladder cells in 200 μl represents 7 × 10^8^ bacterial EVs purified from ≈2.3 mL of bacterial culture. We can only speculate that this is an EV-RNA dose that could be deliverable *in vivo*. Other studies have used significantly higher amounts, i.e., 1–10 μg/mL of bacterial RNA in immune cells (Eberle et al., 2009; Sander et al., 2012; Sha et al., 2014). Furthermore, our study has focused initially upon the acute effects of a single challenge with EV-RNA, and we acknowledge that the evaluation of a chronic effect, perhaps from experiments providing a continuous and longer exposure to EV-RNA, could offer valuable insights.

It is well established that many UPEC virulence factors are responsible for its pathogenicity, including hemolysins, cytotoxins, fimbriae for attachment, flagella for motility, and siderophores, in addition to the ability to form intracellular bacterial communities (IBC) and biofilms (Subashchandrabose and Mobley, 2015). Many *E. coli* virulence factors can be secreted via EVs (Bielaszewska et al., 2017), which provides the opportunity to elicit their effects at distance, in a targeted manner and prior to contact of bacteria live cells with host cells. In this study, we aimed to expand the knowledge of virulence factors involved in the pathogenesis of urinary tract infection, by deciphering the role of the RNA cargo of UPEC EVs. One of the challenges noted were the substantial levels of LPS found in UPEC EVs, which was likely to explain the early transcriptomic response and suppression of IL-1α secretion in bladder cells. This data highlights the importance of understanding the role of EVs as a complex cocktail of factors, which can have unknown interrelations among one another and certainly pleiotropic effects on the host.

## Data Availability Statement

The datasets presented in this study can be found in online repositories. The names of the repository/repositories and accession number(s) can be found below: https://www.ncbi.nlm.nih.gov/geo/, GSE148711.

## Author Contributions

PD-S, JH, SS, AP, and CB designed the experiments and wrote and edited the manuscript. PD-S, JH, and CB performed the experiments and analyzed the results. All authors contributed to the article and approved the submitted version.

## Conflict of Interest

The authors declare that the research was conducted in the absence of any commercial or financial relationships that could be construed as a potential conflict of interest.

## Footnotes

1 https://www.ncbi.nlm.nih.gov/geo/

## Supplementary Material

FIGURE S1 **(A,B,E–G)** Cytokine profiling in bladder cells in response to EV-RNA. Bladder cells were treated with different treatments for 4 h, after which supernatants were assessed by Luminex assays. UPEC, EV-RNA from UPEC strain; Nissle, EV-RNA from Nissle strain; R, EV-RNA obtained from iron limiting culture conditions; RF, EV-RNA obtained from iron sufficient culture conditions; 2, EV-RNA at 2 ng/mL; 0.5, EV-RNA at 0.5 ng/mL; LPS, LPS at 1000 ng/mL delivered with transfection agent unless stated, Free-LPS; LF-Poly I:C, Poly I:C delivered with transfection agent; LF-R848, Resiquimod R848 delivered with transfection agent. Data is plotted as mean and SD of 4 replicates, concentration in *Y*- axis as percentage of control (transfection agent in dotted line). **(C,D)** Viability of cultured bladder cells. Data plotted as Box and whiskers (box represented by upper and lower quartile, line = median, bars = min and max values) of 4 replicates (circles). Stars denote significant differences compared to control (transfection agent only) determined by One-way ANOVA Kruskal–Wallis and Dunn’s posttest (*p* < 0.05).
