# Supplementary figures and images for: Effect of the Extracellular Vesicle RNA Cargo From Uropathogenic Escherichia coli on Bladder Cells
Source: Front Mol Biosci. 2020 Sep 25;7:580913. doi: 10.3389/fmolb.2020.580913 (PMC7546368; doi:10.3389/fmolb.2020.580913)

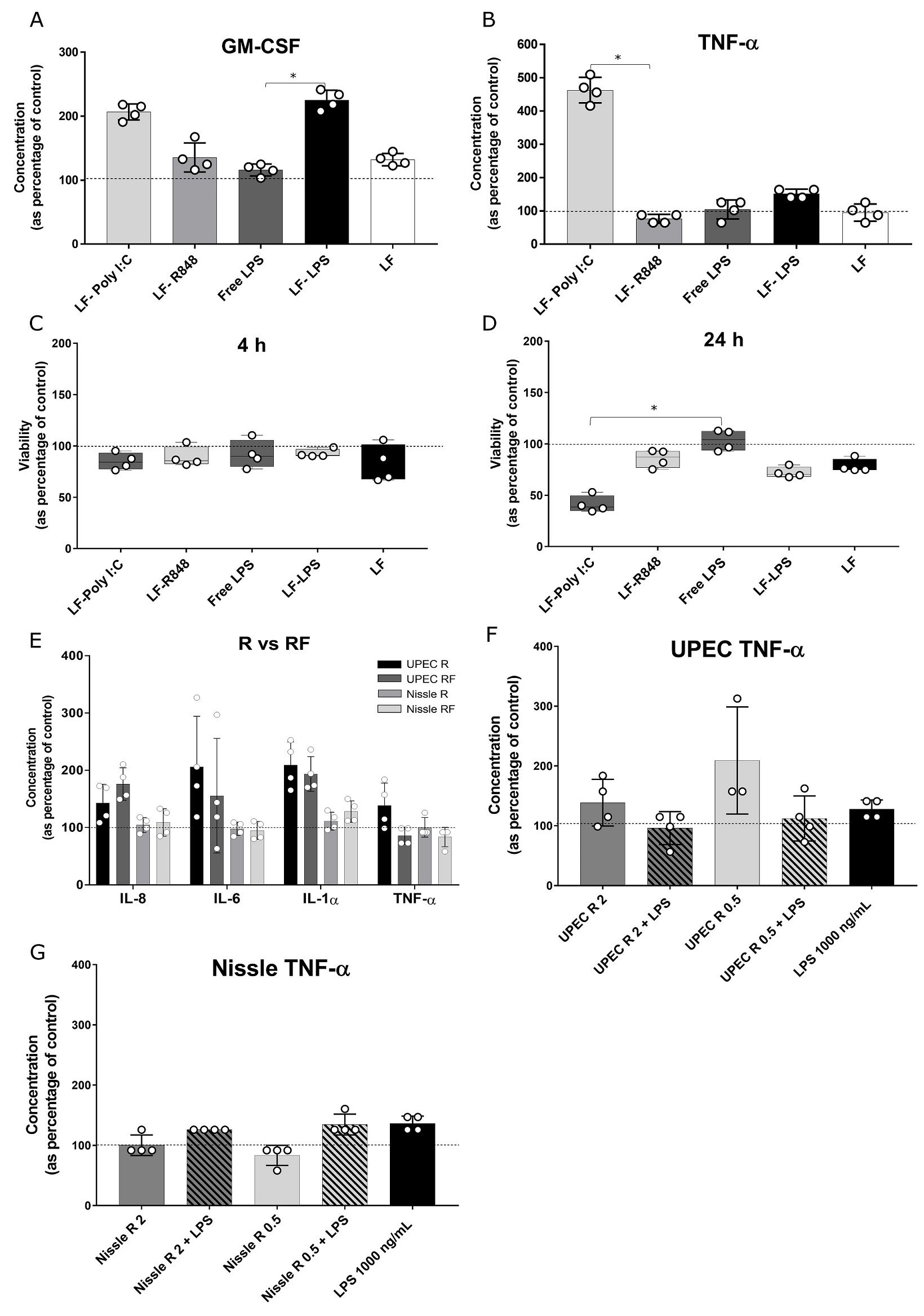

Supplement: FIGURE S1 — (A,B,E–G) Cytokine profiling in bladder cells in response to EV-RNA. Bladder cells were treated with different treatments for 4 h, after which supernatants were assessed by Luminex assays. UPEC, EV-RNA from UPEC strain; Nissle, EV-RNA from Nissle strain; R, EV-RNA obtained from iron limiting culture conditions; RF, EV-RNA obtained from iron sufficient culture conditions; 2, EV-RNA at 2 ng/mL; 0.5, EV-RNA at 0.5 ng/mL; LPS, LPS at 1000 ng/mL delivered with transfection agent unless stated, Free-LPS; LF-Poly I:C, Poly I:C delivered with transfection agent; LF-R848, Resiquimod R848 delivered with transfection agent. Data is plotted as mean and SD of 4 replicates, concentration in Y- axis as percentage of control (transfection agent in dotted line). (C,D) Viability of cultured bladder cells. Data plotted as Box and whiskers (box represented by upper and lower quartile, line = median, bars = min and max values) of 4 replicates (circles). Stars denote significant differences compared to control (transfection agent only) determined by One-way ANOVA Kruskal–Wallis and Dunn’s posttest (p < 0.05). [file Image_1.JPEG]
